# Reduction of Allergenic Potential in Bread Wheat RNAi Transgenic Lines Silenced for *CM3*, *CM16* and *0.28* *ATI* Genes

**DOI:** 10.3390/ijms21165817

**Published:** 2020-08-13

**Authors:** Raviraj M. Kalunke, Silvio Tundo, Francesco Sestili, Francesco Camerlengo, Domenico Lafiandra, Roberta Lupi, Colette Larré, Sandra Denery-Papini, Shahidul Islam, Wujun Ma, Stefano D’Amico, Stefania Masci

**Affiliations:** 1 Department of Agriculture and Forest Science (DAFNE), University of Tuscia, 01100 Viterbo, Italy; rkalunke@gmail.com (R.M.K.); silvio.tundo@unipd.it (S.T.); francescosestili@unitus.it (F.S.); f.camerlengo@unitus.it (F.C.); lafiandr@unitus.it (D.L.); 2 Institute of Plant and Microbial Biology, Academia Sinica, Taipei 11529, Taiwan; 3 Department of Land, Environment, Agriculture and Forestry (TESAF), University of Padova, 35020 Legnaro, Italy; 4 INRAE UR1268 BIA, 44000 Nantes, France; robertinalupi@hotmail.it (R.L.); colette.larre@inrae.fr (C.L.); sandra.denery@inrae.fr (S.D.-P.); 5 Australia China Centre for Wheat Improvement, College of Science Health Engineering and Education, Murdoch University, Murdoch, WA 6150, Australia; S.Islam@murdoch.edu.au (S.I.); w.ma@murdoch.edu.au (W.M.); 6 Institute for Animal Nutrition and Feed, AGES-Austrian Agency for Health and Food Safety, 1220 Vienna, Austria; stefano.d-amico@ages.at

**Keywords:** wheat, RNAi silencing, α-amylase/trypsin inhibitor (ATI), allergy, non celiac wheat sensitivity (NCWS)

## Abstract

Although wheat is used worldwide as a staple food, it can give rise to adverse reactions, for which the triggering factors have not been identified yet. These reactions can be caused mainly by kernel proteins, both gluten and non-gluten proteins. Among these latter proteins, α-amylase/trypsin inhibitors (ATI) are involved in baker’s asthma and realistically in Non Celiac Wheat Sensitivity (NCWS). In this paper, we report characterization of three transgenic lines obtained from the bread wheat cultivar Bobwhite silenced by RNAi in the three ATI genes *CM3*, *CM16* and *0.28*. We have obtained transgenic lines showing an effective decrease in the activity of target genes that, although showing a higher trypsin inhibition as a pleiotropic effect, generate a lower reaction when tested with sera of patients allergic to wheat, accounting for the important role of the three target proteins in wheat allergies. Finally, these lines show unintended differences in high molecular weight glutenin subunits (HMW-GS) accumulation, involved in technological performances, but do not show differences in terms of yield. The development of new genotypes accumulating a lower amount of proteins potentially or effectively involved in allergies to wheat and NCWS, not only offers the possibility to use them as a basis for the production of varieties with a lower impact on adverse reaction, but also to test if these proteins are actually implicated in those pathologies for which the triggering factor has not been established yet.

## 1. Introduction

Wheat (*Triticum* spp.) is one the “big three” cereal crops, together with maize and rice, cultivated worldwide thanks to its adaptability, nutritional value and versatility. This is mostly due to the unique viscoelastic properties of its dough, capable to give rise to a wide range of products, such as bread, pasta, noodles, biscuits, some of which are characteristic of specific geographical areas.

Such properties derive from wheat grain proteins, classified into four major groups based on solvent solubility [1]: albumins (water), globulins (dilute salt solution), prolamins including gliadins (alcohol/water mixture), and finally glutelins, including glutenins (diluted acid or alkaline solutions). At present, gliadins and glutenins are both considered prolamins, because they are soluble in alcohol/water mixtures once glutenins are present in the reduced form.

Gliadins and glutenins make up the gluten, defined as the viscoelastic mass obtained after full flour hydration and washing out of water-soluble components, composed mostly by starch and non-prolamin proteins, namely albumins and globulins (A/G). Among gluten proteins, glutenins play the major role and, in particular, their size and amount are major determinants of dough technological quality [2].

Compared to the gliadins and glutenins, few studies have been carried out on non-prolamins so far. This is probably because their role in flour quality is not as well defined as that of gluten proteins. Nevertheless, A/G constitute around 15−20% of total flour protein [3]. They are a mixture of structural, metabolic and storage proteins [4]. A/G are mostly located in the seed coat, the aleurone cells and the germ; they are relatively scarce in the starchy endosperm [5]. Their amino acid compositions are relatively well balanced because of higher lysine content as compared to the prolamin fraction. Predominant A/G components such as α-amylase/trypsin inhibitors (ATI), serpins and purothionins have multiple functions; indeed, they serve as nutrient reserves for the germinating embryo and as inhibitors of insects and fungal pathogens before germination [6].

Wheat proteins can cause different adverse reactions, some of which are better characterized, such as in Celiac Disease (CD), Wheat Allergies including Food Allergy to Wheat (FAW), Wheat-Dependent Exercise-Induced Anaphylaxis (WDEIA), or in Baker’s Asthma (BA) [7]. Differently, the role of wheat components in Irritable Bowel Syndrome (IBS) or Non Celiac Wheat/Gluten Sensitivity (NCWS or NCGS, respectively) is still not clear. In particular, this can be deduced by the use of the two names, NCWS or NCGS to describe a pathology that includes both gastrointestinal and non-gastrointestinal symptoms caused by wheat ingestion, but that excludes CD and FAW. Because specific serological markers are not present so far, this is actually a self-reported condition, whose diagnosis is based on double-blind placebo-controlled wheat challenge [8]. This situation makes it even more difficult to establish the triggering factor, that initially was identified in gluten, mostly for analogies with CD, but that at present indicates rather ATI or fermentable oligosaccharides disaccharides monosaccharides and polyols (FODMAPs), reason why it is currently preferred to use the name NCWS, rather than NCGS. Since the prevalence worldwide is in the range 0.6−13%, it is important to identify the real culprit of such pathology.

Wheat ATI are among the putative triggering factors of NCWS and are unquestionably involved in BA, the most common occupational respiratory disease in Western countries, affecting about 10% of flour workers [9]. Moreover, this class of wheat proteins seems involved in some wheat-related food-allergies, and, to a minor extent, with WDEIA [10]. In this regard, recently, Tundo et al. [11] tested three heterologously expressed ATI proteins, named *CM3*, *CM16* and 0.28 in basophils degranulation assay against human sera of patients with FAW. Although all the three proteins induced degranulation, the most effective one was *CM3*, accounting for the important role played by this protein in triggering allergic reactions. Moreover, *CM3* has an important role in innate immune response, at least in monocytes, macrophages, and dendritic cells [12,13].

Most ATI proteins belong to the so-called CM protein fraction of wheat, because they are soluble in chloroform and methanol solutions [14]. Three classes of ATI are typically described, that correspond to monomeric, dimeric and tetrameric forms, with different specificities against various heterologous **α**-amylases. In particular, the 12 kDa monomeric inhibitors, also known as 0.28 proteins, are encoded by genes on the short arms of the group 6 chromosomes; the 24 kDa homodimeric inhibitors, also known as the 0.19 and 0.53 proteins, are encoded by genes on the short arms of the group 3 chromosomes; the third group is constituted of the 60 kDa heterotetrameric inhibitors composed of one copy of either CM1 or CM2, encoded by genes on chromosomes 7D or 7B, plus one copy of either CM16 or CM17, encoded by genes on chromosomes 4B or 4D, plus two copies of *CM3*, also encoded on chromosomes 4B or 4D [15].

The availability of genotypes with reduced amounts of ATI proteins might allow either the development of novel wheat cultivars with a lower triggering potential, or a better understanding of the role of these components in such wheat-related pathologies. Recently, our group [16] has reported the application of CRISPR-Cas9 silencing technology to silence *CM3* and *CM16* ATI genes in durum wheat, whereas in this paper we report the development and the extensive characterization, including the allergenic potential and qualitative evaluations, of wheat lines derived from the bread wheat cultivar Bobwhite, in which *CM3*, *CM16* and *0.28* ATI genes have been silenced by RNAi.

## 2. Results

### 2.1. RNAi Transformed Wheat Lines Show Silencing of ATI Genes During the Kernel Development Stages

In total, 1669 immature embryos of *Triticum aestivum* L. cv. Bobwhite were co-transformed using three RNAi constructs (*pRDPT-CM3*, *pRDPT-CM16* and *pRDPT-0.28*) and the plasmid pUBI::BAR. Eight T_0_ independent transgenic lines resistant to Bialaphos were obtained which represents 0.44% transformation efficiency. The presence of the transgenes was verified by PCR analysis on genomic DNA from regenerated plants (data not shown). No significant differences in morphology or growth were observed between the “null” genotype that had lost the transgene by segregation (null-segregant), and the untransformed plants (data not shown), thus we have used the untransformed plant (indicated as either BW or WT) for the experiments.

In order to get homozygous transgenic line, T_1_ progenies from T_0_ plants containing all three transgenes as well as the *bar* marker gene were used in segregation analysis. PCR analyses were performed on DNA extracted from half-seeds using primers specific for the *CM3*, *CM16*, *0.28* and *bar* transgenes. Lines containing all four transgenes were used for further seeds production to obtain homozygous transgenic lines showing silencing of the three target genes. In T_4_ generation, homozygous transgenic line plants were obtained and used for the analyses, in comparison with WT.

Relative gene expression analyses were performed on the lines 10-10a, 22-2 and on the Bobwhite control plants to investigate the reduction of transcripts of the genes encoding *CM3*, *CM16* and *0.28* subunits during kernel development stages. In this case, we could not use line 24-1 because we did not get enough developing grains to extract mRNA.

**Figure 1:**
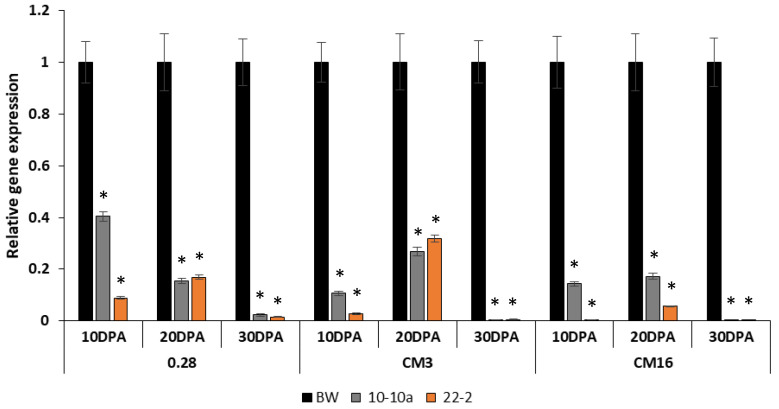
Expression analysis of *CM3*, *CM16*, *0.28* α-amylase/trypsin inhibitors (ATI) genes by qRT-PCR in different caryopses growth stages (10, 20 and 30 days post anthesis (DPA)) in the bread wheat cv Bobwhite (BW) and RNAi-silenced lines 10-10a and 22-2. The relative gene expression is reported as the fold increase in the transcripts compared to Bobwhite control plants. Standard error is shown above each bar, along with asterisk to indicate where the value differed significantly (*p* < 0.05).

Data obtained from RNA extracted at 10 Days Post Anthesis (DPA) showed an immediate reduction in the first development stages of all transcripts investigated. The decrease in the gene expression of the *CM3*, *CM16* and *0.28* subunits was more evident in line 22-2, but it was also significant for in line 10-10a. The reduction of the transcripts, in fact, ranged from 80% to almost 100% in line 22-2 for all the genes analyzed, whereas in line 10-10a, the reduction ranged from 60% to 90%; a similar result was obtained from the analysis carried out with RNA extracted at 20 DPA with a slight increase in the transcripts compared to 10 DPA. The almost total silencing of ATI genes was quite clear at 30 DPA for both RNAi lines (Figure 1).

### 2.2. Targeted Proteins CM16, 0.28 and CM3 in the Transgenic Lines Are Accumulated to a Lower Extent with Respect to the Control

Differential expression of the grain proteins across the silenced lines and in comparison with the parental cultivar Bobwhite, were analyzed using a 4-plex iTRAQ experiment. A total of 3915 distinct peptides have been detected at >95% confidence level. The total number of detected proteins was 794 with ≥1 peptide matching, while the threshold of ≥2 peptides matching downsized the protein numbers to 550. Compared to the parental cultivar, 80, 80 and 74 proteins showed differential expression in the silenced lines 24-1, 22-2 and 10-10a, respectively. Overall, the protein expression changes across the silenced lines compared to the parental cultivar were consistent.

The results demonstrated that the expression of the targeted three ATI, namely *CM16* (TraesCS4B01G328000), *0.28* (TraesCS6D01G000200) and *CM3* (TraesCS4B01G328100) were reduced remarkably in the silenced lines with proper statistical significance (Table S1). In the silenced line 24-1, all the three target proteins expression reduced more than the other two silenced lines. Proteins *CM16*, *0.28* and *CM3* showed 91-, 79- and 72-fold reductions in expression, respectively, in the silenced line 24-1, compared to the parental cultivar (Table S1). On the other hand, in lines 22-2, and 10-10a, those protein’s expressions were reduced by 25, 24 and 25 folds; and 17, 23 and 24 folds, respectively. In addition, *CM17* which has 85% sequence similarity with CM16, showed 59, 28 and 29 folds reduced expression in silenced lines 24-1, 22-2 and 10-10a, respectively (Table S1). Furthermore, some other closely related proteins, namely dimeric *α*-amylase inhibitor, trypsin inhibitor CMc, Bowman–Birk type trypsin inhibitor and thaumatin-like xylanase inhibitor also showed reduced expression in the silenced lines which ranged from a 5- to a 14-fold reduction (Table S1). Several other trypsin inhibitor proteins also demonstrated reduced expression but were excluded from the interpretation due to the higher *p* value than the threshold of 0.05.

### 2.3. Some of Non-Target Proteins Show Differential Accumulation in Silenced Lines Compared to the Control

The expression patterns of the all identified glutenin and gliadin proteins were analyzed across the silenced lines and in comparison with the parental cultivar. A total of 21 proteins of these groups showed differential expression across the silenced lines (Table S2), although only four of them were statistically significant, considering the cut off *p* value of 0.05. The high molecular weight glutenin subunits (HMW-GS) identified as 1Dx1.6t, likely corresponding to 1Dx5, showed a 64-, 22- and 17-fold reduction in expression in silenced lines 24-1, 22-2 and 10-10a, respectively (Table S2). However, another HMW-GS corresponding to 1Dy10 subunit (Uniprot accession No. A9YSK3) showed 19.23-fold reduced expression in the line 10-10a only. On the other hand, an s-type LMW-GS (Uniprot accession No. F8SGN6) showed 3.63-, 0.14- and 4.29-fold reductions in expression in silenced lines 24-1, 22-2 and 10-10a, respectively. Furthermore, another LMW-GS (Uniprot accession No. R4JBK0), but of m-type, showed 6.73- and 7.31-fold reductions in expression in silenced lines 22-2 and 10-10a, respectively, although it did not show significant reduction in line 24-1. Differential expression of all the gliadin proteins and other LMW and HMW-GSs were statistically non-significant (Table S2).

However, several non-target proteins demonstrated differential expression (by both over expression and reduced expression) in the silenced lines compared to the parental cultivar (Table S3). An uncharacterized protein (Uniprot accession No. A0A3B6RB62) whose molecular function is nutrient reservoir activity, showed reduced expression by 44-, 17- and 15-fold in the silenced lines 24-1, 22-2 and 10-10a, respectively. The other major down regulated proteins in the silenced lines included Avenin-like b2, Puroindoline b, AAI domain-containing protein, grain softness proteins and farinin protein (Table S3). On the other hand, an uncharacterized protein (Uniprot accession No. W5GLX4) whose molecular function is ATPase activity, showed a 99-fold increased expression in all the three lines compared to the parental cultivar (Table S3). The other major upregulated proteins in all the silenced lines included DUF89 domain-containing protein, 60S acidic ribosomal protein P2B, WHy domain-containing protein and Eukaryotic translation initiation factor 5A.

### 2.4. ATI Reacting with Anti-ATI Antibody Was Lower in All the Three Transgenic Lines Compared to Bobwhite Control

In order to assess whether the transformation with silencing constructs of bread wheat could lead to a reduction of ATI content in kernels, we performed indirect enzyme-linked immunosorbent assay (ELISA) experiments using a polyclonal antibody against α-amylase inhibitors on A/G fraction.

**Figure 2:**
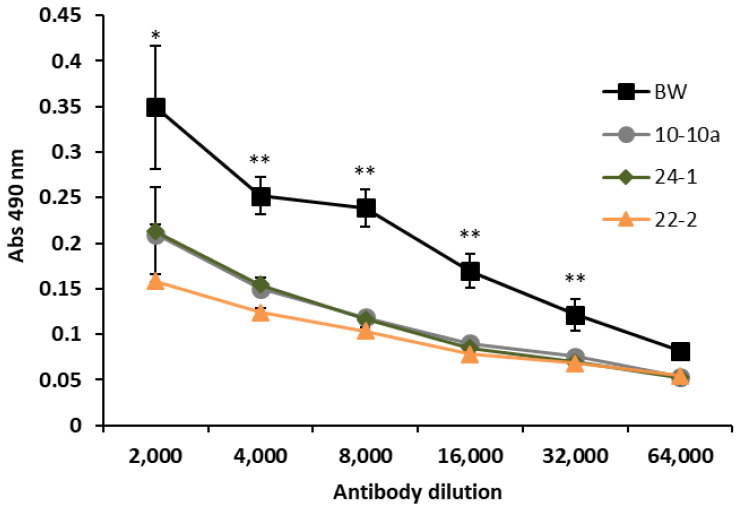
Enzyme-linked immunosorbent assay (ELISA) performed on albumins and globulins (A/G) fraction of Bobwhite control plants (BW)and transgenic lines with polyclonal anti ATI antibody. Values represent the average of the absorbance at 490 nm ± standard error of three biological and three technical replicates. All data were subjected to ANOVA analysis and Tukey test. ** indicates difference between RNAi transgenic lines and Bobwhite control plants at *p* < 0.01 level of significance, * indicates *p* < 0.05 level of significance.

The ELISA analysis revealed that the amount of ATI reacting with anti-ATI antibody was lower in all the three transgenic lines compared to Bobwhite control plants (Figure 2). The levels of antigen were significantly different between transgenic lines and Bobwhite control plants in all primary antibody dilutions, except for the lower ratio (1:64,000), for which no difference was observed.

### 2.5. Sera of Wheat Allergic Patients Show a Lower Reactivity to the Transgenic Lines

**Figure 3:**
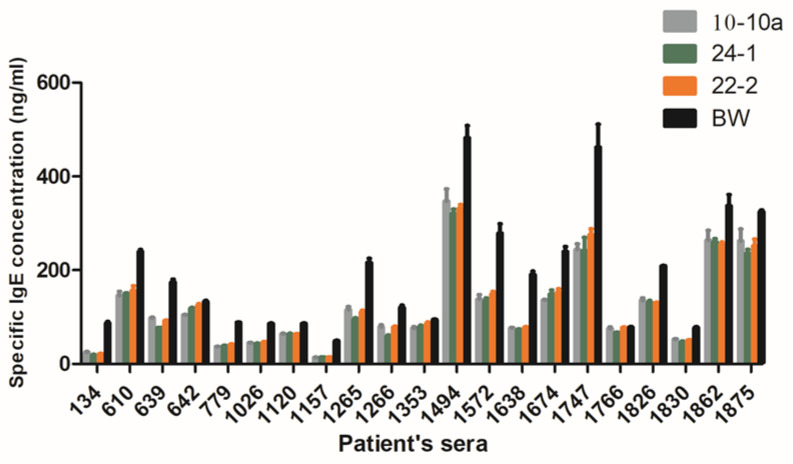
Human IgE binding to salt soluble proteins from the three RNAi transgenic lines and from the Bobwhite control plants (BW), detected in the sera from 22 allergic patients by fluorescent (F)-ELISA assay. Results are expressed as the means of specific IgE concentration. For major clarity, ANOVA analysis is reported in the Supplementary File.

All the twenty-two sera selected for their content in IgE specific to the A/G fraction of the bread wheat cultivar Recital reacted with all the genotypes. A broad variability (from 10 to 500 ng/mL) in the IgE binding capacity between genotypes was measured, in all cases the IgE binding of Bobwhite was higher than that of RNAi silenced lines. In general, more than half of the sera showed lower reactions to the silenced lines with respect to Bobwhite (Figure 3). Two-way ANOVA was performed to investigate the effect of the three ATI silenced lines with respect to Bobwhite and the effect of sera on IgE binding capacity (In Supplementary Files). The two factors (genotype and sera) and their interaction were significant (*p* < 0.05). The genotypes account for 7.27% of the total variance, whereas sera account for 86.30% of the total variance. This latter result is related to the well-known variation existing between individual reactions against the same antigen.

### 2.6. Transgenic Wheat Lines Do Not Affect Trypsin Inhibition Activity, but Mostly α-Amylase Activity

The presence of trypsin inhibitors is health-related because they are involved in several gastro-intestinal and non-intestinal disorders. Thus, we have performed a measure of TIA (trypsin inhibitor activity) in the wheat lines in which specific *α*-amylase and trypsin inhibitors were silenced.

In Table 1, TIA is reported both as absolute and normalized values according to the amount of soluble protein tested. Noteworthy, the total protein amount in the silenced lines almost doubled that of Bobwhite, and this is reflected in the amount of soluble proteins as well, including ATI. This is likely a compensation effect, already known for other wheat genotypes in which specific gene expression is suppressed either by transgenesis [17] or by gene deletions [18]. TIA results were coherent with protein amounts, being increased in silenced lines, but, when normalized, significant lower values were observed in line 22-2 and 10-10a.

This result was also confirmed in the progress of trypsin inhibition by using increasing sample extract volumes. In Figure S1 it is shown that, in the three silenced lines, the maximum of trypsin inhibition is reached with lower sample extract volumes with respect to the reference genotype Bobwhite, indicating that a higher amount of trypsin inhibitors might be present in the transgenic lines. MALDI TOF spectra (Figure S2) clearly indicated lower signal intensities in the molecular weight range of ATI and the peak at about 15.5 kDa of *CM3* disappeared almost completely. Previously [19], it has been shown that a broad variety of proteins are bound to trypsin and thus play a role for trypsin inhibition. Furthermore, *α*-amylase/subtilisin inhibitor was slightly increased in modified samples as seen in Table S1. Nevertheless, mentioned facts cannot explain completely variations in TIA. Other proteins, probably uncharacterized ones like W5GLX4 and A0A453HBR5 (Table S3) might play a key role for the measured trypsin inhibition levels because they increased strongly (up to 99%). An uncharacterized protein, A0A077RSX3_WHEAT, was found to inhibit trypsin as well [19]. However, since the other results we have obtained clearly indicate that the three target genes have been silenced and absence of most ATI, as shown also by LC-MS/MS data, the results got about TIA indicate that the three target genes do not have a large trypsin inhibition activity, but they likely inhibit *α*-amylase activity. Furthermore, aggregation state of ATI might be important for their biological functionality, but this characterization was not performed.

### 2.7. Influence of Silencing of the Three Target ATI Genes on Predictive Quality Parameters

Thousand kernel weight (TKW) and coleoptile length are two parameters correlated with wheat yield and have been measured to test if possible pleiotropic effects of the silencing may influence such important aspect. No significant differences were detected in TKW between RNAi transgenic lines and Bobwhite control plants (Figure S3). The coleoptile lengths of the WT and transgenic lines were compared at two different timepoints: 5 and 7 days post germination (DPG). No significant difference was observed in any of the two time points (Figure S4). The absence of significant differences in both the tested parameters showed that yield was not affected by the silencing of the three ATI genes.

**Figure 4:**
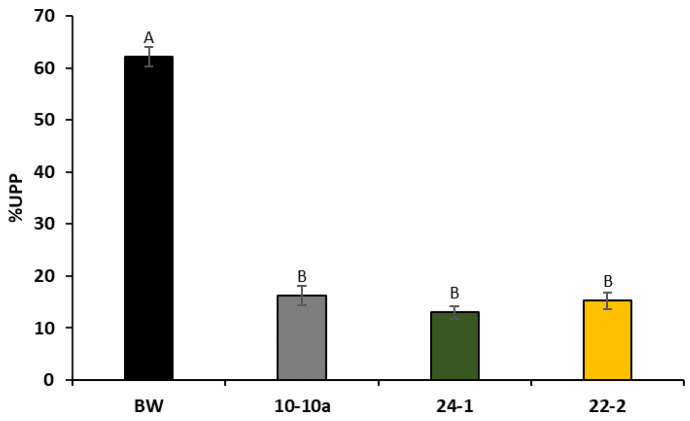
Determination of percentage of unextractable polymeric proteins (%UPP) in Bobwhite control plants (BW) and the three RNAi transgenic lines. Values represent the average of three biological and three technical replicates. All data were subjected to ANOVA analysis. Letters above the histograms correspond to ranking of Tukey test at 0.95 confidence and *p* < 0.01 level of significance.

Since higher amounts of flour are needed to perform rheological tests, we used percentage of unextractable polymeric proteins (%UPP) micro-test to predict gluten quality of RNAi-silenced plants. Figure 4 reports the histograms representing the %UPP values found for Bobwhite control plants and the three transgenic lines. ANOVA analysis showed that there is a dramatic decrease in the %UPP value in all silenced lines, as compared to Bobwhite control plants.

Afterwards, we analyzed the electrophoretic pattern of glutenin subunits, whose structure influences the formation of glutenin polymers, the major parameter determining %UPP.

**Figure 5:**
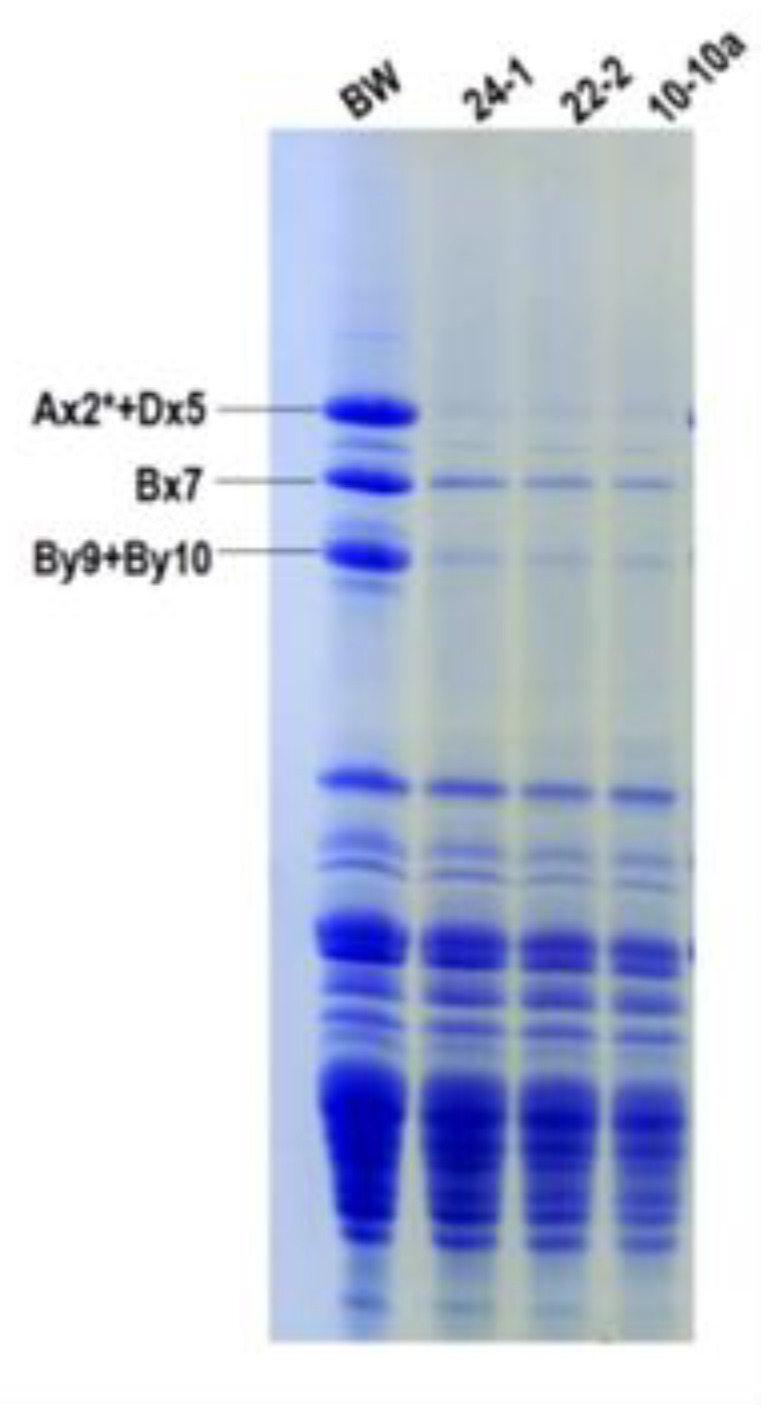
SDS-PAGE of total wheat kernel protein subunits extracted from the three RNAi transgenic lines and Bobwhite control plants. High molecular weight glutenin subunits (HMW-GS) identification is reported.

The SDS-PAGE pattern of total kernel proteins is reported in Figure 5. Although already indicated by gene expression analyses and LC-MS/MS results, this gel clearly shows that HMW-GS have been an off-target of RNAi silencing, in particular the *Glu-D1* coded pair 5+10, mostly responsible of qualitative characteristics of bread wheat. This observation could explain the decrease in %UPP.

**Figure 6:**
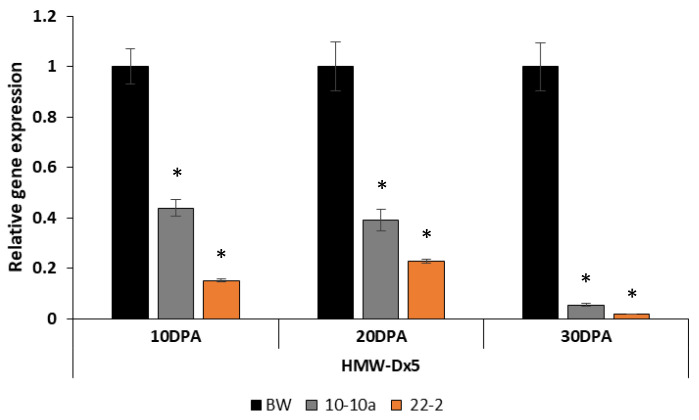
Expression analysis of *HMW-GS Dx5* gene by qRT-PCR in different caryopses growth stages (10, 20 and 30 DPA) in the control line BW and RNAi-silenced lines 10-10a and 22-2. The relative gene expression is reported as the fold increase in the transcripts compared to Bobwhite control plants. Standard error is shown above each bar, along with asterisk to indicate where the value differed significantly (*p* < 0.05).

The expression analysis of *HMW-GS Dx5* gene was tested and revealed a significant reduction of Dx5 transcripts already at 10 DPA, especially for the line 22-2. At 30 DPA, the amount of the *Dx5* transcripts was drastically reduced in both transgenic lines, approximately 90% less (Figure 6). In this case also, only two transgenic lines have been analyzed because of the lack of enough developing grains of line 24-1 (Figure 6).

To determine if non-targeted silencing of *HMW-GS* was due to possible homologies among *ATI* and *HMW-GS* genes, we made a bioinformatics comparison among the three target *ATI* genes and the genes coding for the *HMW-GS* genes present in cultivar Bobwhite, that are Ax2*, *Dx5*, *Bx7*, *By9* and *Dy10*.

The analysis allowed us to identify the presence of a conserved region in *0.28* and *HMW-GS By9* and *Dy10* (TGCTGCCAGCAGCT), that might explain the off-target silencing for at least of these two subunits. Moreover, the alignment between *0.28* and *HMW-GS Dy10* showed a further homology of some nucleotides separated from the conserved region by a guanine pair (Figure S5). The conserved sequence has been submitted to a further analysis with PlantGRN (http://plantgrn.noble.org), containing information on plant transcriptional regulation. By using the psRNATarget and ppsRNAi tools, the presence of siRNA and miRNA is suggested. psRNATarget highlights 16 miRNA sequences with sequence homologies with 0.28 and HMW-GS Dy10. By using the ppsRNAit tool, 18 possible siRNA have been identified (Figure S6).

## 3. Discussion

Although RNAi is a transgenic procedure, it can be used as a proof of concept before developing new genotypes with non-transgenic techniques. Regardless of the procedure used, in fact, any new genetic combination can potentially give rise to unintended effects. Silencing of specific genes can be pursued by exploiting natural variation present in genetic resources, or by generating pseudogenes by classical or advanced mutagenesis, but all the classical methods require time consuming procedures that, in case undesirable unintended effects are produced, make the work performed useless.

The possibility to develop new wheat genotypes accumulating a lower amount of proteins potentially or effectively involved in such pathologies, not only offers the possibility to use them as a basis for the creation of wheat varieties with a lower impact on adverse reaction, but also to test if these proteins are actually implicated in those pathologies for which the triggering factor has not been established yet.

Recently we have obtained durum wheat lines in which the ATI genes *CM3* and *CM16* have been silenced by CRISPR-Cas9 multiple editing [16], and that are now under multiplication and characterization. Differently, in the present work, we report the characterization, in terms of protein and gene expression, of allergenic potential, and of parameters related to technological performances of bread wheat lines in which three genes coding for ATI proteins, namely *CM3*, *CM16* and *0.28*, involved in BA and likely in NCWS, have been silenced by RNAi.

We have obtained different transgenic lines showing an effective decrease in the target genes, and in the corresponding protein products. Very interesting was the observation that, differently from what was expected, the assay of trypsin inhibition (TIA) showed higher levels for the transgenic lines. These findings are confirmed by the study of Call et al. [20], which found no significant correlation between ATI concentration and TIA. As already mentioned, besides ATI, other non-gluten proteins probably play a key role in trypsin inhibition.

Whatever the explanation, such a result clearly highlights the role of the three target genes in allergic reactions, because the silenced lines result in effectively less reactivity with respect to the untransformed line, when tested with sera of patients allergic to wheat. Especially *CM3* might be the most important factor in triggering allergies, as suggested also by Tundo et al. [11], that, after testing heterologously produced *CM3*, *CM16* and *0.28* in basophils degranulation assay against human sera of patients with FAW, found that *CM3* was the most effective, giving results comparable to those of a whole soluble protein extract.

This plant material is available for testing the role of the silenced proteins in triggering either allergic reactions or NCWS, because it does not show significant differences in terms of yield, although, from the technological point of view, it is rather poor, due to the non-target effect on HMW-GS, mostly influencing rheological properties of wheat doughs. Another possible explanation might reside in epigenetic silencing mechanisms due to the elevated number of *Dx5* promoters in ATI transgenic lines. A silencing mechanism, known as homology-dependent gene silencing (HDGS), can arise when multiple copies of the same or homologous sequences are introduced in a genome [21,22]. Jauvion et al. [23] distinguished two types of HDGS, based on the stage at which it occurs: the first is the transcriptional gene silencing (TGS) that is associated with transcription and promoter modification; the second one is the post-transcriptional gene silencing (PTGS), which occurs after the formation of mRNA. In our transgenic plants, the RNAi constructs and the endogenous *HMW-GS* gene have a high homology in the promoter regions, so the activation of a TGS mechanism is plausible, that can lead to the methylation of the promoter or coding regions of endogenous *HMW-GS*. Similar results were previously observed in transgenic plants, in which a non-symmetrical methylation caused the silencing of endogenous genes [22]. If the explanation of such an untargeted effect resides in epigenetic silencing mechanisms due to the high number of Dx5 promoters in ATI transgenic lines, this could be circumvented by back-crossing the transgenic lines with the WT genotype, in order to decrease the number of Dx5 promoters and possibly restore the proper HMW-GS composition.

## 4. Materials and Methods

### 4.1. Plant Material and Transformation

RNAi constructs preparation, wheat transformation by the biolistic procedure and the selection of the transgenic lines were carried out as previously described by Sestili et al. [24]. Briefly, three vectors *pRDPT-CM3*, *pRDPT-CM16* and *pRDPT-0.28* were prepared for RNAi cassettes of *ATI* genes *CM3*, *CM16* and *0.28*, respectively. These genes were amplified using cDNA template generated from RNA extracted from immature embryos (28 Days Post Anthesis, DPA) of *Triticum aestivum*. L. cv. Bobwhite. The cDNA synthesis was performed by using the Quanti-Tect Reverse Transcription Kit (Qiagen, Hilden, Germany) following the manufacturer instructions. The vector pRDPT contains promoter and terminator of the *Dx5* high molecular weight glutenin subunit gene and intron of starch branching enzymes of class II (*SBEIIa*). Sense and antisense sequences of the target region of *ATI* gene were inserted in the plasmid pRDPT separated by the *SBEIIa* intron with help of restriction sites introduced in the oligonucleotides (denoted in primer name). Restriction sites *SalI* and *ClaI* were used for the insertion of sense sequences, whereas *XhoI* and *XbaI* were used for antisense sequences. The following primers were used (sequences are listed in Table S4) to amplify the three target genes: for *CM3* sense sequence iRNA_Sal-CM3-F and iRNA ClaI-CM3-R; *CM3* anti-sense sequence: iRNA XhoI-CM3-F, and iRNA XbaI-CM3-R. For *CM16* sense sequence: iRNA SalI-CM16-F, 5′- and iRNA ClaI-CM16-R; *CM16* Anti-sense sequence: iRNA XhoI-CM16-F and iRNA XbaI-CM16-R. For 0.28 sense sequence: iRNA Sal-AAI0.28-F and iRNA ClaI-0.28 R; *CM0.28* Anti-sense sequence: iRNA XhoI-0.28_F, and iRNA XbaI-0.28-R.

The immature embryo derived calli of the bread wheat cultivar Bobwhite were co-bombarded with the three RNAi plasmids (*pRDPT-CM3*, *pRDPT-CM16* and *pRDPT-0.28*) and the plasmid *pUBI::BAR* (carrying the *bar* gene as selection marker) mixed in a 3:1 molar ratio following the biolistic procedure.

The presence of the construct in Bialaphos-resistant plants and their progeny was verified by PCR on genomic DNA obtained from young leaves of the regenerated plants [25] using specific primer pairs (sequences are listed in Table S4) for *CM3* (iRNA_Sal-CM3-F, and Intron R), *CM16* (Dx5_Promo 2F and iRNA ClaI-CM16-R), 0.28 (Dx5_Promo 2F and iRNA ClaI-0.28 R) and *bar* gene (UBI-49F and BAR 2). The *actin* gene amplification was used as positive control to confirm the presence of genomic DNA template in PCR reaction, using actin-specific primer pair: Actin 77F and Actin 312R. PCR amplifications were carried out according to the procedure for GoTaq PCR Master Mix (Promega, Madison, WI, USA) and the amplification conditions were as follows: 1 cycle at 95 °C for 2 min; 35 cycles at 95 °C for 1 min, 60 °C for 1 min, and 72 °C for 1 min; and a final 3-min step at 72 °C. The PCR products were loaded into 1.2% (*w*/*v*) agarose gels and subjected to electrophoresis followed by visualization under UV light (data not shown).

Three homozygous transgenic lines (named as 24-1, 22-2 and 10-10a) showing silencing of the three genes have been advanced to the T_4_ generation, thus T_4_ plants were used for the reported analyses, in comparison with the control untransformed line (WT).

### 4.2. Quantitative Analyses of ATI Transcripts by qRT-PCR

All the lines were cultivated in growth chambers in the same conditions. The qRT-PCR was performed on total RNA extracted from the caryopses of the three RNAi lines along with the wild type line at 10 DPA, 24 DPA and 30 DPA, using the Spectrum Plant Total RNA kit (Sigma-Aldrich, St. Louis, MO, USA) and following manufacturer’s instructions. First strand complementary DNA was synthesized using the Quanti-Tect Reverse Transcription Kit (Qiagen, Hilden, Germany) according to the manufacturer’s instructions from one μg of total RNA of each sample. The experiment was carried out with the same primer used for amplification of target genes reported above. For *Dx5* transcripts, the following primers were used: Dx5F2: 5′-GACCAACAGCTCCGAGACA-3′ and Dx5R2: 5′-GTATGAAACCTGCTGCGGAC-3′. The *actin* gene was used as reference gene. The analyses were performed in the CFX 96 Real-Time PCR Detection System device (Bio-Rad, Hercules, CA, USA). The reactions were prepared in a final volume of 15 μL, consisting in 7.5 μL SsoAdvUniver SYBR GRN SMX (Bio-Rad, Hercules, CA, USA), 0.5 μM of each primer and 1 μL of cDNA, following a three-step PCR protocol: 95 °C for 30 sand 39 cycles at 95 °C for 10 s, 60 °C for 25 s and 72 °C for 15 s. Three biological replicates with three technical replicas were carried out for each reaction and the maximum difference accepted between Ct values of technical replicates was 0.5. The relative quantification normalized to the reference gene was performed using the 2^-ΔΔCt^ method.

### 4.3. Protein Analysis by iTRAQ

Total proteins were obtained by crashed kernels by extraction with a buffer containing 0.5 M Tris-HCl buffer pH 8.8, 1% dithiothreitol (DTT) in 50% propanol for 1 h at room temperature, and precipitating with 4 volumes of cold acetone, by repeating this latter step three times, in order to obtain pure wheat proteins free from salts and other reagents. Eventually proteins present in the pellet were freeze dried. Before iTRAQ analysis, total protein samples were further acetone precipitated, reduced, alkylated, trypsin digested [26] and labelled according to the manufacturer’s iTRAQ protocol (SCIEX, Framingham, MA, USA). The samples were then labelled using the iTRAQ reagents (Table S5).

All labelled samples were combined to make a pooled sample. Peptides were desalted on a Strata-X 33 μm polymeric reversed phase column (Phenomenex, Torrance, CA, USA) and dissolved in a buffer containing 2% acetonitrile 0.1% formic acid, before separation by High pH on an Agilent 1100 HPLC system using a Zorbax C18 column (2.1 × 150 mm) (Agilent Technologies, Palo Alto, CA, USA). Peptides were eluted with a linear gradient of 20 mM ammonium formate, 2% ACN to 20 mM ammonium formate, 90% ACN at 0.2 mL/min. The 95 fractions were concatenated into 12 fractions and dried down. Each fraction was analyzed by electrospray ionization mass spectrometry using a Thermo UltiMate 3000 nanoflow UHPLC system (Thermo Scientific, Waltham, MA, USA) coupled to a Q Exactive HF mass spectrometer (Thermo Scientific, Waltham, MA, USA). Peptides were loaded onto an Acclaim™ PepMap™ 100 C18 LC Column, 2 μm particle size x 150mm (Thermo Scientific, Waltham, MA, USA) and separated with a linear gradient of water/acetonitrile/0.1% formic acid (*v/v*). Amount of pooled sample loaded on the mass spectrometer: 9 μg.

#### 4.3.1. Data Analysis

All the raw MS data were analyzed using ProteinPilotTM software versions 5.0 (AB Sciex) for protein identification and quantification. ProteinPilot applies a hybrid approach to integrate de novo sequencing with a traditional database searching approach in identifying proteins. Firstly, high-confidence small peptide sequence segments are identified, called taglets; taglets are then used to improve the downstream database searching procedure [27]. ProteinPilot is a well-established protein identification algorithm in the proteomics community. Spectral data were analyzed against the database “UniProt Taxonomy: *Viridiplantae* (Green plants), Version: August 2019”. The number of the sequences in the database was 8,344,090. iTRAQ impurity was automatically corrected following the protocol provided by ABI Sciex for ProteinPilot.

#### 4.3.2. Quality Control and Filtering

The statistical analysis was performed at protein levels. The protein identifications were filtered following several criteria:

1. All the artificially introduced false positive identifications by ProteinPilot’s FDR analysis were filtered;
2. Proteins identified below 95% confidence by ProteinPilot standard (identification score < 1.3) were discarded from downstream analysis;
3. Proteins unused for quantification were discarded from the analysis, including those having discordant peptide type, quantification signal too weak, and with low confidence;
4. The proteins that have at least one unique peptide assigned were accepted.

### 4.4. SDS-PAGE

The electrophoretic control of lines was performed on the albumin and globulin fraction, containing ATI, including the CM proteins, in all lines obtained, as well as on total gluten proteins.

A/G extraction was performed on pools of mature grains, that were crushed and mixed with extraction buffer containing 0.05M phosphate buffer/0.1M NaCl, pH 7.8 (3 g: 80 mL). After centrifugation at 8000 *g* for 15 min at 4 °C, the supernatant was precipitated with acetone; the pellet, corresponding to the A/G proteins was then rinsed two times. Protein concentrations of the A/G proteins were determined with the ‘Bio-Rad Protein assay’ kit [28]. The A/G fraction was solubilized in Tris-HCl buffer, pH 6.8, containing 2% SDS, 10% glycerol, 0.1% 2-mercaptoethanol and bromophenol blue. SDS-PAGE was performed using stacking gels (T = 3.75%; C = 2.67%) to concentrate the proteins and running gels (T = 15%; C = 0.9%) to separate the proteins based on their molecular weight.

SDS-PAGE was performed using the Mini-PROTEAN Tetra Vertical Electrophoresis Cell (Bio-Rad) and the gels were 1.0 mm thick. For each sample, 15 μg of protein extract was loaded into the gel that was run at a constant voltage of 200V until the bromophenol blue dye front disappeared.

Total protein subunits were extracted from 10 mg of flour of RNAi transgenic lines and Bobwhite control plants in a 1:10 ratio *w*/*v* in 70 mM Tris-HCl, pH 6.8, 2% SDS, 10% glycerol, 0.02% γ-pyronine and 1% DTT, stirring for 1 h at room temperature. After centrifugation at 13,000 *g* for 10 min, the extracts were quantified with the ‘Bio-Rad Protein assay’ kit and 10 µg of protein was used for SDS-PAGE analysis through SE 600 Hoefer (Fisher Scientific, Waltham, Massachusetts, USA) device. The main gel was T = 11 and C = 1.28, while the stacking gel was T = 3.75 and C = 2.67. The electrophoresis was performed at 40 mA per gel at 10 °C and stopped as the tracking dye reached the bottom of the gel.

In both cases, gels were stained according to Neuhoff et al. [29].

### 4.5. Immunological Tests

#### 4.5.1. Sera from Wheat Allergic Patients and Controls

Sera from patients with clinically documented FAW were obtained with the informed consent of the patients and with Ethic Committee approbation. Eight control sera were obtained from healthy volunteers. Twenty-two sera were selected based on their concentrations in specific IgE for albumins/globulins measured by F-ELISA (Table S6). Sera were obtained from the Biological Resource Center (BB-0033-00038) of Clinical Immunology and Allergy Service of Angers University Hospital (France) with the informed consent of the patients.

#### 4.5.2. Indirect ELISA with Anti-ATI Antibodies

The enzyme-linked immunosorbent assay (ELISA) was performed for quantifying ATI in the A/G fraction of the WT and transgenic lines. For each genotype, three replicates of each sample (A/G fraction) were tested, except for the two transgenic lines 24-1 and 22-2, whose samples were tested in duplicate. The wells on microtiter plate (ELISA plate 82.1581.100, Sarstedt, Nümbrecht, Germany) were coated with the A/G fractions at 5 μg/mL in 100 mM carbonate (pH 9.6) for 1h at room temperature. After three washes with PBS-0.05% Tween 20, the plate was blocked with PBS-4% milk for 1h at 37 °C. The microplate was washed three times with PBS-0.05% Tween 20 and then incubated for 1h at 37 °C with a serial dilution of primary antibody from 1:2000 to 1:64,000 in PBS-2% milk; a monoclonal antibody against ATI, properly developed in mouse at the INRA, Nantes (France). After three washes with PBS-0.05% Tween 20, the plate was incubated for 1h at 37 °C with the secondary antibody (anti-rabbit IgG labelled with the enzyme HRP). After three additional washes with PBS-0.05% Tween 20, the colorimetric substrate for HRP OPD (o-Phenylenediamine) in 0.05M citrate buffer plus H_2_O_2_ were introduced to have a yellow-orange product detectable at 490 nm. The OPD reaction was stopped after 30 min with H_2_SO_4_ 4N. Three biological and three technical replicates were used. All data were subjected to ANOVA analysis by using SYSTAT12 software (Systat Software Incorporated, San Jose, CA, USA). When significant F values were observed, a pairwise analysis was carried out by the Tukey Honestly Significant Difference test (Tukey test).

#### 4.5.3. ELISA Test with Sera of Allergic Patients

Sera obtained from 22 patients with allergy to wheat were characterized by ELISA using salt soluble protein extracts (Table S6), as described by Battais et al. [30]. Specific IgE concentrations against the soluble fraction from Bobwhite and RNAi lines were determined by fluorescent (F)-ELISA on white 384-well plates (NUNC 460372, Fisher Scientific, Waltham, MA, USA), using alkaline-phosphatase-conjugated goat anti-human IgE and 4-MUP as a substrate (Sigma A3525 and M3168, respectively, Saint-Quentin Fallavier, France) as described by and Lupi et al. [31]. Patient sera were diluted from 1:10 to 1/50 according to the serum reactivity.

The concentration of specific IgE binding to the antigen was calculated using a standard curve, as described by Bodinier et al. [32]. Three replicates were performed for each serum. Data are presented as mean values with their standard errors.

Statistical analyses were performed using GraphPad Prism 5.02 software (GraphPad Software, La Jolla, CA, USA), and *p* values below 0.05 were considered significant. Analyses of variance (Two-way ANOVA) with subsequent Bonferroni’s multiple-comparisons tests between means of the Bobwhite vs. RNAi lines were performed.

### 4.6. Trypsin Inhibition Assay

Trypsin inhibition was measured according to Call et al. [19]. An amount of 2 g of flour was extracted with 10 mL 150 mM NaCl solution containing 0.02 M phosphate buffer (pH 7.0) by magnetic stirring for 5 min at room temperature followed by centrifugation (4000 rpm for 10 min.). Equilibration was performed for 20 min at 37° C and enzyme/substrate ratio of 1:100 was used. For determination of the linear region four to seven different sample volumes of salt-water extracts were tested, depending on the strength of inhibition. Each sample was extracted three times, and each extract was measured in duplicate with an optimized sample volume. TIA values were expressed in mg/kg and mg/g protein based on protein concentration determined by the Bradford assay. The total protein content was analyzed by the Dumas combustion method with a Dumaster equipment from Büchi (Uster, Switzerland) and a conversion factor of 5.7 was applied.

MALDI TOF MS of salt-water extracts was performed according Call et al. [20] after purification with C18 ZipTips (Millipore, Merck, Germany).

### 4.7. Parameters Related to Yield and Calculation of the Percentage of Unextractable Polymeric Proteins (%UPP)

Thousand kernel weight (TKW) was used as a yield indicator. It was calculated by weighing and counting the kernels. Besides, coleoptile length was used as a parameter to measure seedling vigor and growth. It was measured at 5 and 7 days after imbibition (DPI) by using a scale. For both parameters, two independent experiments were performed. Data were subjected to ANOVA analysis.

The percentage of unextractable polymeric proteins (%UPP) was determined according to Gupta et al. [2]. Soluble glutenin polymers were extracted from 10 mg of flour by using 50% ACN 0.05% TFA in a 1:10 (*w*/*v*) ratio. The samples were left under stirring for 30 min at room temperature and subsequently centrifuged for 15 min at 10,000 *g*. The supernatant was filtered through 0.45-μm filters (Ultrafree-MC, centrifugal filters, PVDF, Millipore Corp., Billerica, MA) by centrifugation at 16,900 g for 10 min. The insoluble polymers were extracted by adding to the pellet from the previous extraction 50% ACN 0.05% TFA, in a 1:10 ratio. The extraction of insoluble polymers was carried out by sonication (Vibra cell, Sonics Materials, INC), with a 5-mm probe with the following settings: tune 50, output control 25 for 20 sec. The supernatant was filtered through PVDF 0.45-μm filters by centrifugation at 16,900 g for 10 min. For the Size Exclusion Chromatography (SEC) analysis of soluble and insoluble polymers, HPLC System Gold 126 NM (Beckman Coulter) was used with management and acquisition software 32 Karat 7.0, equipped with UV detector Spectra Series UV150 (Thermo Separation Products) set at 214 nm and chromatographic 30 cm column molecular exclusion BioSep-SEC-S 4000 (Phenomenex) with an internal diameter of 4.6 mm. The samples were injected using 100-μL loop connected to a six-way valve, elution occurred under isocratic conditions using 50% ACN 0.05% TFA eluent, at a flow rate of 1 mL/min for a duration of the stroke chromatography of 10 min. The column was kept at the temperature of 30 °C. Three biological and three technical replicates were used. All data were subjected to ANOVA analysis by using SYSTAT12 software (Systat Software Incorporated, San Jose, CA, USA). When significant F values were observed, a pairwise analysis was carried out by the Tukey Honestly Significant Difference test (Tukey test).

### 4.8. Bioinformatic Comparison of HMW-GS Gene Sequences

The NCBI (National Centre for Biotechnology Information https://www.ncbi.nlm.nih.gov.) data bank was used to compare *0.28* (accession number AJ223492.1), *CM3* and *CM16* (accession numbers AY436554.1 and X17573.1, respectively), with the following *HMW-GS* genes: *Dx5* (accession number DQ907161.1), *Dy10* (accession number X12929.2), Ax2* (accession number M22208.2), *Bx7* (accession number BK006773.1) e *By9* (accession number X61026.1).

Alignment was performed with the software ClustalOmega (https://www.ebi.ac.uk/Tools/msa/clustalo/r).

## Figures and Tables

**Table 1:** Concentration of total protein, salt-water soluble proteins and trypsin inhibition activity (TIA).

Sample	Total Protein (g/100 g)	Soluble Protein (g/100 g)	TIA (mg/Kg)	TIA (mg/g Soluble Protein)
Bobwhite	10.08 ± 0.03 a	1.28 ± 0.13 a	107.89 ± 1.92 a	8.43 ± 0.15 c
24-1	21.10 ± 0.07 b	2.40 ± 0.32 b	207.54 ± 3.14 d	8.65 ± 0.13 c
22-2	22.18 ± 0.05 c	2.78 ± 0.05 b	170.75 ± 3.50 b	6.13 ± 0.12 a
10-10a	20.83 ± 0.06 b	2.52 ± 0.14 b	197.82 ± 2.71 c	7.86 ± 0.10 b

Small letters indicate homogeneous subgroups based on ANOVA (*p* ≤ 0.05) and post hoc test according to Scheffe.

## Supplementary Materials

Supplementary Materials can be found at https://www.mdpi.com/1422-0067/21/16/5817/s1.

## Abbreviations

A/G	Albumins and Globulins
ATI	α-Amylase/Trypsin Inhibitors
BA	Baker’s Asthma
BW	Bobwhite
CD	Celiac Disease
DPA	Days Post Anthesis
DPG	Days Post Germination
DPI	Days Post Imbibition
FAW	Food Allergy to Wheat
FODMAPs	Fermentable Oligosaccharides Disaccharides Monosaccharides And Polyols
HDGS	Homology Dependent Gene Silencing
HMW-GS	High Molecular Weight Glutenin Subunits
IBS	Irritable Bowel Syndrome
NCGS	Non Celiac Gluten Sensitivity
NCWS	Non Celiac Wheat Sensitivity
PTGS	Post-Transcriptional Gene Silencing
qRT-PCR	Quantitative Real-Time PCR
TGS	Transcriptional Gene Silencing
TIA	Trypsin Inhibitor Activity
TKW	Thousand Kernel Weight
%UPP	Percentage of Unextractable Polymeric Proteins
WDEIA	Wheat-Dependent Exercise-Induced Anaphylaxis
WT	Wild Type (the untransformed control genotype corresponding to BW)
